# Effects of acetaldehyde-induced DNA lesions on DNA metabolism

**DOI:** 10.1186/s41021-019-0142-7

**Published:** 2020-01-06

**Authors:** Haruka Tsuruta, Yuina Sonohara, Kosuke Tohashi, Narumi Aoki Shioi, Shigenori Iwai, Isao Kuraoka

**Affiliations:** 1 Department of Chemistry, Faculty of Science, Fukuoka University, 8-19-1 Nanakuma, Jonan-ku, Fukuoka, 814-0180 Japan; 2 Division of Chemistry, Graduate School of Engineering Science, Osaka University, 1-3 Machikaneyama, Toyonaka, Osaka 560-8531 Japan

**Keywords:** Acetaldehyde, DNA lesion, DNA metabolism

## Abstract

**Background:**

Acetaldehyde, produced upon exposure to alcohol, cigarette smoke, polluted air and sugar, is a highly reactive compound that is carcinogenic to humans and causes a variety of DNA lesions in living human cells. Previously, we reported that acetaldehyde reacts with adjacent deoxyguanosine residues on oligonucleotides, but not with single deoxyguanosine residues or other deoxyadenosine, deoxycytosine, or thymidine residues, and revealed that it forms reversible intrastrand crosslinks with the dGpdG sequence (GG dimer).

**Results:**

Here, we show that restriction enzymes that recognize a GG sequence digested acetaldehyde-treated plasmid DNA with low but significant efficiencies, whereas restriction enzymes that recognize other sequences were able to digest such DNA. This suggested that acetaldehyde produced GG dimers in plasmid DNA. Additionally, acetaldehyde-treated oligonucleotides were efficient in preventing digestion by the exonuclease function of T4 DNA polymerase compared to non-treated oligonucleotides, suggesting structural distortions of DNA caused by acetaldehyde-treatment. Neither in vitro DNA synthesis reactions of phi29 DNA polymerase nor in vitro RNA synthesis reactions of T7 RNA polymerase were observed when acetaldehyde-treated plasmid DNA was used, compared to when non-treated plasmid DNA was used, suggesting that acetaldehyde-induced DNA lesions inhibited replication and transcription in DNA metabolism.

**Conclusions:**

Acetaldehyde-induced DNA lesions could affect the relative resistance to endo- and exo-nucleolytic activity and also inhibit in vitro replication and in vitro transcription. Thus, investigating the effects of acetaldehyde-induced DNA lesions may enable a better understanding of the toxicity and carcinogenicity of acetaldehyde.

## Introduction

Most carcinogens damage DNA and generate mutations in the genome [1]. For example, aflatoxin B1, which is considered one of the most important fungal mycotoxins in human food, is altered into a reactive form via metabolic processes in the liver. This reactive form induces aflatoxin-DNA adducts by reacting with guanine in DNA and causes guanine to thymine trans-version mutations [2, 3]. As such induced mutation profiles are associated with a characteristic mutation of the p53 tumor suppressor gene, aflatoxin B1 is considered a contributory cause of liver cancer in many tropical regions, where hepatocellular carcinoma is a major cause of cancer death. The cancer-causing chemical benzo[a]pyrene, found in coal tar and tobacco smoke, is metabolized in human cells to benzo[a]pyrene diol epoxide, which forms covalent DNA adducts with guanine and induces mutations in cells [4, 5]. Carcinogens also include various forms of radiation. Ultraviolet (UV) radiation directly reacts with DNA and generates bulky DNA lesions such as cyclobutane pyrimidine dimers (CPD) and 6–4 pyrimidone photoproducts (6-4PP) [6, 7]. If left unrepaired, they may induce mutations and eventually cause skin cancers.

Induced DNA lesions, associated with cancer, inborn diseases and aging, interfere with replication, leading to mutations and cell death [1]. In addition, such DNA lesions may also interfere with transcription, by inhibiting elongation via RNA polymerase and reducing transcription and/or mutation of transcripts [8]. Therefore, biochemical risk assessment studies of chemicals that induce DNA lesions in DNA metabolism are important.

Acetaldehyde is carcinogenic to humans. It is classified by the International Agency for Research on Cancer as a substance for which sufficient evidence indicating its carcinogenicity in humans is available [9]. Much of the evidence for carcinogenicity of acetaldehyde has been obtained via animal experiments. Acetaldehyde is a small, highly reactive compound that occurs naturally in various plants, ripe fruits, and vegetables [10]. It is a key raw material used to produce a wide range of chemical substances. Consuming alcoholic beverages, smoking cigarettes, breathing polluted air, and ingesting sugars can lead to a buildup of acetaldehyde in the body [11]. Humans are constantly exposed to acetaldehyde, contact with which is seemingly unavoidable in our environment.

Thus, acetaldehyde, a highly reactive compound that is anticipated to be a human carcinogen, reportedly causes a variety of DNA lesions in living cells [12, 13]. The direct product of the reaction between acetaldehyde and deoxyguanosine is the Schiff base type adduct, *N*^2^-ethylidenedeoxyguanosine. This product is relatively unstable and may be stabilized via chemical reduction (or biochemical reduction) of the Schiff base to form the stable product, *N*^2^-ethyldeoxyguanosine. However, most major acetaldehyde-induced DNA lesions have very little effect on replication, because replicative DNA polymerases can bypass them in a non-mutagenic manner. On the contrary, during transcription elongation, lesions may block RNA synthesis by RNA polymerases.

Previously, we reported that acetaldehyde reacts with adjacent deoxyguanosine residues on oligonucleotides, but not with single deoxyguanosine residues or any other deoxyadenosines, deoxycytosine or thymidine residues. It forms reversible intrastrand crosslinks with the GG dimer, which resembles the UV-induced dimer lesions, CPD and 6-4PP [14]. The current study indicated that acetaldehyde-treated plasmid DNA, which remained incomplete digested by restriction enzymes that recognize a GG sequence, was digested by restriction enzymes that recognize other sequences. Moreover, acetaldehyde-treated oligonucleotide DNA was relatively efficient in preventing digestion by exonucleolytic activity. The use of the above plasmid DNA did not result in DNA or RNA synthesis reactions. An investigation of the effect of acetaldehyde-treated DNA on DNA metabolism may help clarify the toxicity and mutagenicity of acetaldehyde.

## Materials and methods

### Enzymes and chemicals

Phi29 DNA polymerase, restriction enzymes (*Mlu*CI, *Hae*III, *Msp*I, *Hha*I) and 6x Gel loading Dye were purchased from New England Biolabs (NEB: Ipswich, MA, USA). T4 DNA polymerase and random primers were obtained from Takara (Shiga, Japan). T7 RNA polymerase and reverse transcriptase were from TOYOBO (Osaka, Japan). The RNase inhibitor was purchased from Wako (Osaka, Japan). Fast SYBR Green Master Mix was obtained from Life Technologies (Carlsbad, CA, USA), and acetaldehyde (extra pure reagent) was obtained from Nacalai Tesque (Kyoto, Japan).

### Acetaldehyde and UV treatment

Plasmid (pBluescript II SK (−) containing the T7 promoter; Stratagene, La Jolla, CA, USA: pBSII) DNA templates were purified using a QIAGEN Midi Kit (QIAGEN, Hilden, Germany). For the purpose of acetaldehyde treatment, DNA templates were incubated with 1 M acetaldehyde at 37 °C for 1 h. Although the boiling point of acetaldehyde is 20.2 °C, we used 37 °C that is an optimal temperature for commonly used enzymes. For UV treatment, UV-light (254 nm, 450 J/m^2^) was used. Next, the templates were purified using Sephadex G-25 columns according to the manufacturer’s instructions (GE Healthcare, Amersham, Buckinghamshire, UK).

### Nuclease assays

**Fig. 1:**
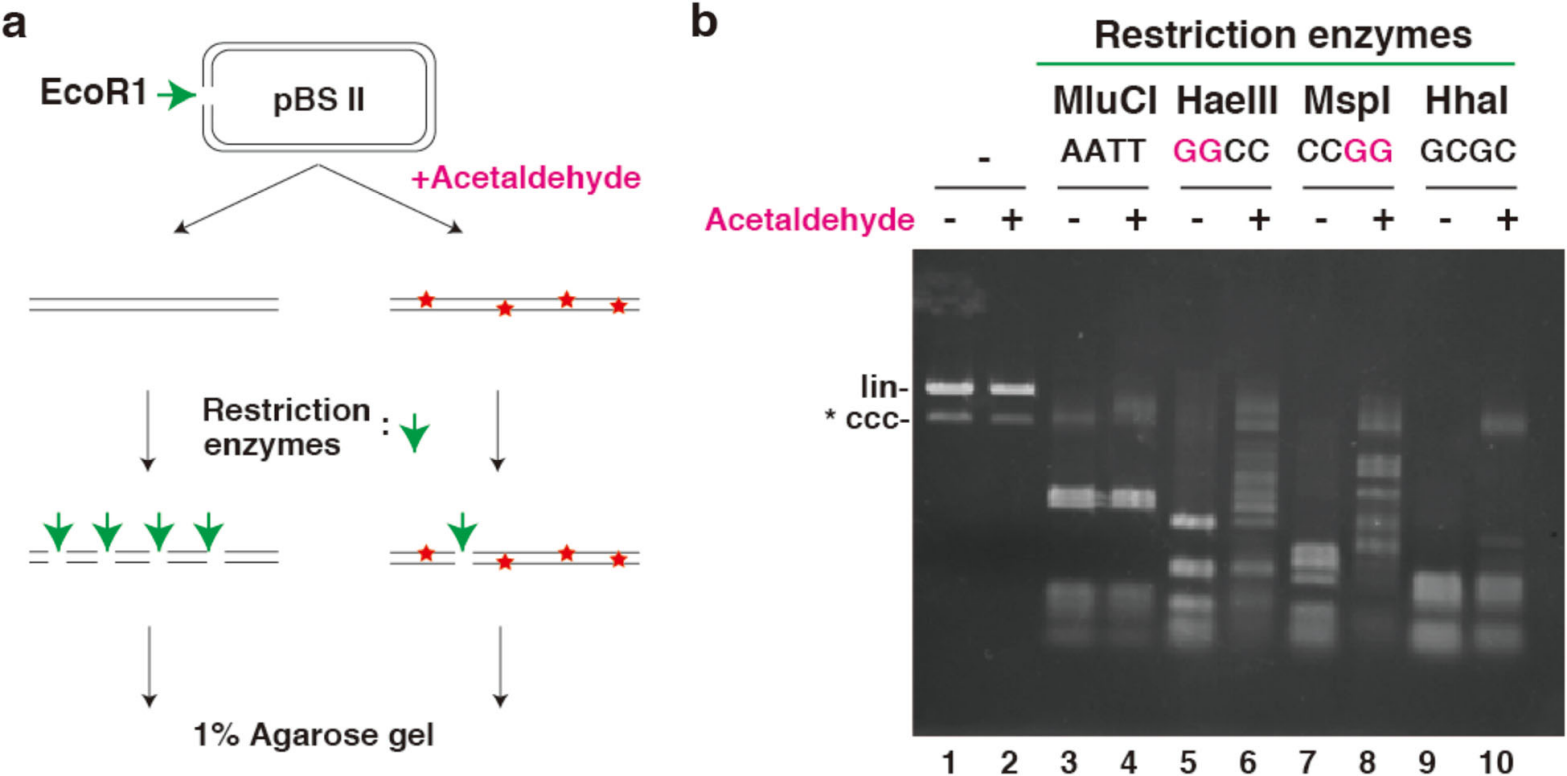
Endonucleolytic digestion of acetaldehyde-treated plasmids. **a** In the absence of DNA damage, the indicated restriction enzymes generated digested DNA fragments from *Eco*RI-digested pBS DNA templates. If acetaldehyde induced damages GG sequences in DNA are present, the resulting GG lesions are resistant to digestion by restriction enzymes, and full-digested DNA fragments will not be detected on agarose gel. **b** Agarose gel (1%) demonstrating the presence of the GG lesion. *Eco*RI-digested pBSII DNA templates (lanes 1 and 2) were digested with restriction enzymes *Mlu*CI (lanes 3 and 4), *Hae*III (lanes 5 and 6), *Msp*I (lanes 7 and 8) and *Hha*I (lanes 9 and 10). Acetaldehyde-treated *Eco*RI-digested pBSII DNA templates (lanes 2, 4, 6, 8, and 10). The mobility of the linear (lin) *Eco*RI-digested pBSII DNA templates and the covalently closed circular (ccc) undigested pBSII DNA templates is indicated along the side of the gel

For endonucleolytic digestion, *Eco*RI-digested pBSII DNA templates were treated with acetaldehyde and digested using the indicated restriction enzymes according to the manufacturer’s instructions. Reactions were terminated by the addition of 6x Gel loading Dye and the sample DNA substrates were subjected to 1% agarose gel, and visualized by ethidium bromide staining (Fig. 1a).

**Fig. 2:**
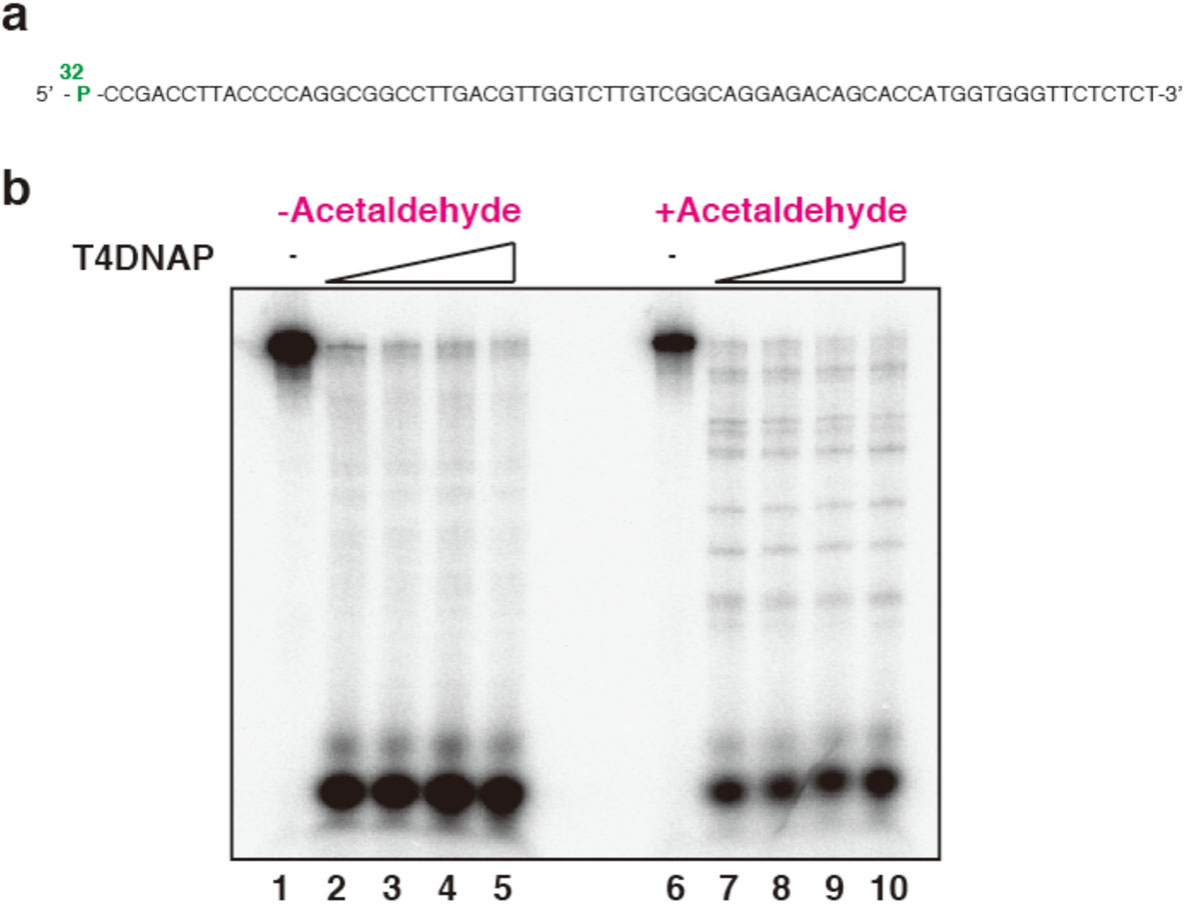
Exonucleolytic digestion in acetaldehyde-treated oligonucleotides. **a** Schematic drawing of ^32^ P -labelled 70-mer oligonucleotide. **b** Action of the exonuclease function of T4 DNA polymerase on an oligonucleotide containing acetaldehyde induced DNA lesions. Non-treated oligonucleotides (lanes 1–5) and acetaldehyde-treated oligonucleotides (lanes 6–10) were digested with increasing amounts of T4 DNA polymerase (0, 0.3, 0.75, 1.5, and 3 units) in the absence of deoxynucleoside triphosphates at 37 °C for 30 min

For 3′ exonucleolytic digestion, 5′-^32^P end-labeled 70-mer oligonucleotides (Fig. 2a) were treated with acetaldehyde and the samples were incubated with different amounts of exonucleases as indicated in the figure legends. T4 DNA polymerase was assayed in 10 μL reaction mixtures containing 50 mM NaCl, 10 mM Tris-HCl (pH 7.9), 10 mM MgCl_2_ and 1 mM dithiothreitol at 37 °C for 30 min. Reactions were terminated by the addition of 10 μL of stop solution containing 95% formamide, 20 mM EDTA, 0.025% bromphenol blue and 0.025% xylene cyanol. Oligonucleotide fragments were separated by electrophoresis on a denaturing 12.5% polyacrylamide gel, dried and visualized by a Fuji FLA-7000 phosphorimager (Fujifilm, Tokyo, Japan).

### Phi29 DNA polymerase-replication assay

For in vitro replication assay, 40 μL reactions of 1 μg pBSII and 100 pmol random primer were conducted under incubation at 95 °C for 5 min at RT for 20 min. DNA samples were treated with acetaldehyde. Next, 50 μL reactions containing 200 ng DNA template, 0.2 mM dNTP mixture (dATP, dCTP, dGTP and dTTP), 100 ng/ml bovine serum albumin and 5 units Phi29 DNA polymerase in buffer (50 mM Tris–HCl, pH 8.0, 10 mM (NH_4_)_2_SO_4_, 10 mM MgCl_2_ and 4 mM dithiothreitol) were conducted under incubation at 37 °C for the indicated incubation times. Replication products were analyzed via 1% agarose gel electrophoresis and quantified using NIH Image software.

### T7 RNA polymerase-transcription assay

For in vitro transcription assay [15], 50 μL reactions containing 100 ng DNA template, 4 mM NTP mixture (ATP, CTP, GTP, and UTP) and 5 units thermo T7 RNA polymerase in buffer (40 mM Tris–HCl, pH 8.0, 50 mM NaCl, 8 mM MgCl2, 5 mM dithiothreitol and 20 units RNase inhibitor) were incubated at 37 °C for 1 h. RNA transcripts were purified using an RNeasy Mini Kit (QIAGEN) with RNase-Free DNase (QIAGEN) according to the manufacturer’s instructions. Next, cDNAs were generated from purified RNA samples using primer 2440–2421 (5′-gcggccaacttacttctgac-3′) and ReverTra Ace reverse transcriptase (TOYOBO) according to the manufacturer’s instructions. Real-time quantitative PCR (rt-qPCR) was performed on a StepOne System (Life Technologies) using Fast SYBR Green Master Mix (Life Technologies) with primers 2140–2159 (5′-tatcagcaataaaccagcca-3′) and 2440–2421 (5′-gcgg ccaacttacttctgac-3′) to ensure the appearance of a single product peak (301 bp) from mock mixtures in the melting curve analysis. Each reaction was run in triplicate, and the data were plotted as ΔRn versus cycle number.

## Results

### Nucleolytic activity on acetaldehyde-induced DNA lesions

Recently, we reported that acetaldehyde may induce GG intra crosslink lesions in reversible reactions [14]. To investigate the effects of the lesions on DNA metabolism, such as replication and transcription, we first analyzed the inhibitory effects exerted by the digestion of endonucleolytic and exonucleolytic enzymes.

*Eco*RI-digested linear pBSII DNA templates were treated with acetaldehyde and digested using the indicated restriction enzymes and the samples were loaded on a 1% agarose gel. As expected there was no change in migration on a 1% agarose gel between non- and acetaldehyde-treated DNA templates (Figs. 1b, lanes 1 and 2). *Mlu*CI is able to recognize an AATT sequence, and a pBSII DNA template contains 12 AATT sequence sites. Thus, the *Mlu*CI-digested DNA sample (−), which was not treated with acetaldehyde on the gel, indicated a complete DNA digestion pattern (Fig. 1b, lane 3). The *Mlu*CI-digested DNA sample (+), which was treated with acetaldehyde, also showed the same pattern as that of the sample that was not treated with acetaldehyde. On the other hand, *Hae*III and *Msp*I are able to recognize GGCC and CCGG sequences, respectively. The DNA pattern produced by enzymes digesting non-treated DNA is shown in Fig. 1b, lanes 5 and 7. When the acetaldehyde-treated DNA template was used, however, these enzymes produced a partial digestion pattern. *Hha*I, which recognizes a GCGC sequence, produced an almost complete DNA digestion pattern in either non-acetaldehyde or acetaldehyde-treated DNA (Fig. 1b, lane 9 and 10). The results indicated that acetaldehyde induced DNA lesions prevented restriction enzyme mediated digestion. As acetaldehyde-plasmid treatment induced GG intra crosslinked lesions in DNA, we used these procedures in the experiments which followed.

DNA lesions are known to prevent exonuclease activity. Thus, acetaldehyde treated oligonucleotides were incubated with T4 DNA polymerase, which functions as a processive 3 to 5 exonuclease in the absence of deoxynucleoside triphosphates. This enzyme has been used to detect bulky DNA lesions such as UV- and cisplatin-induced DNA lesions [16, 17]. Non-treated oligonucleotides were completely digested by the exonucleolytic activity of T4 DNA polymerase (Fig. 2b, lanes 2–5), but acetaldehyde treated oligonucleotides produced partial resistance to this activity (Fig. 2b, lanes 7–10). This result indicated that acetaldehyde induces exonucleolytic resistant DNA lesions in oligonucleotides. We could not determine the damaged site in the oligonucleotide sequence (Fig. 2a), suggesting the chemical instability of the lesions as previously reported [14].

### DNA replication reaction in acetaldehyde-treated plasmids

**Fig. 3:**
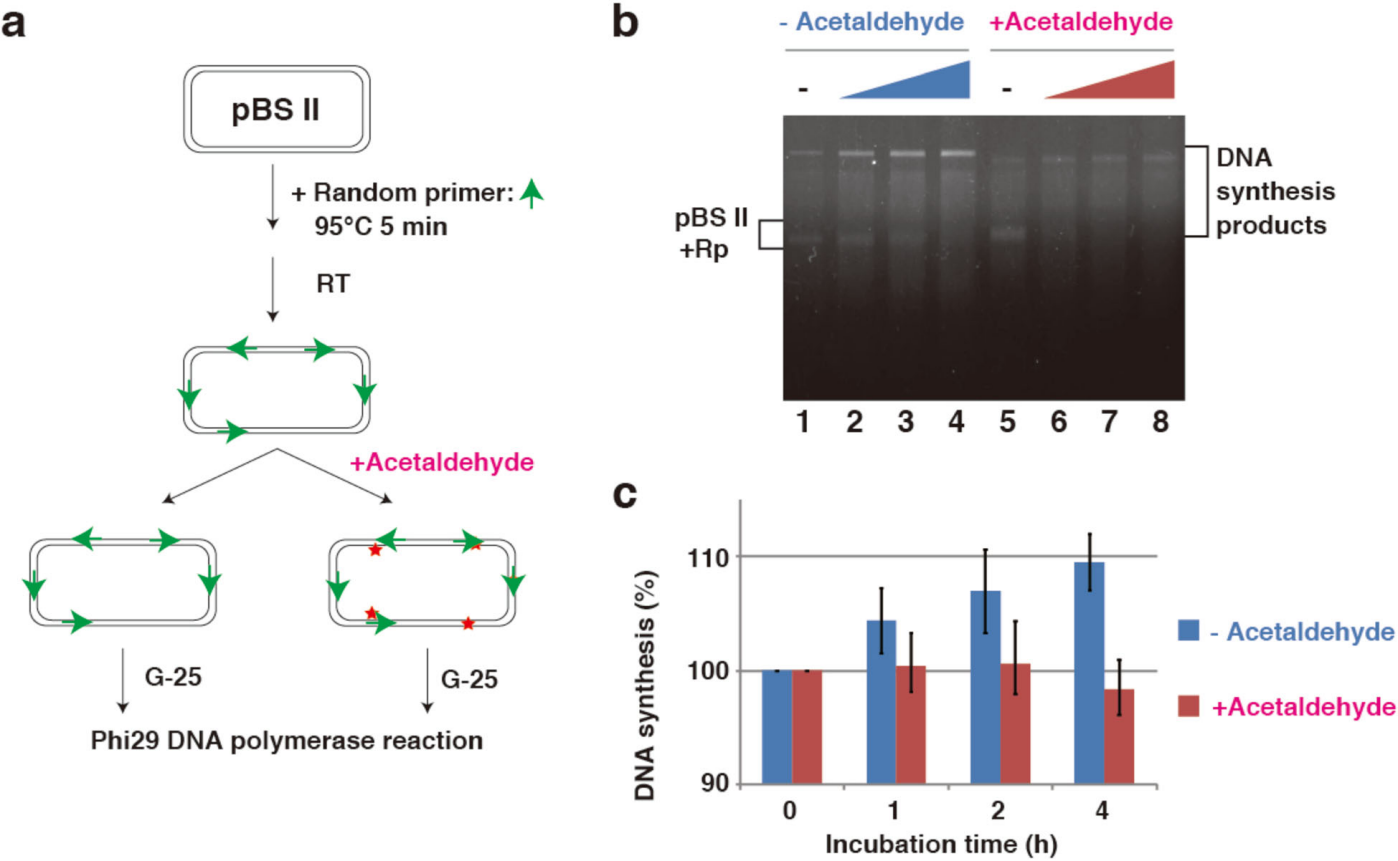
DNA replication reaction in acetaldehyde-treated plasmids. **a** In the absence of DNA damage, phi29 DNA polymerase and random primers generate new DNA synthesis products from the template. If acetaldehyde damages DNA, the resulting lesions inhibit DNA synthesis, as phi29 DNA polymerase cannot synthesize new DNA products from damaged templates, and products will not be detected. **b** Agarose gel (1%) demonstrating the presence of an acetaldehyde-induced lesion. The phi29 DNA polymerase and non-acetaldehyde treated DNA template/random primer complexes (lane 1) or acetaldehyde treated DNA template/random primer complexes (lane 5) were incubated for the indicated times (0,1, 2, and 4 h: lanes 1–4 or lanes 5–8). Rp is random primers and triangles are incubation time. **c** Quantification of DNA synthesis products via 1% agarose gel analysis (b)

Next, we investigated whether DNA polymerase synthesizes DNA strands on acetaldehyde-treated DNA templates. For this propose, we used phi29 DNA polymerase, a replicative polymerase from the *Bacillus subtilis* phage phi29 [18]. This polymerase shows strand displacement and processes synthesis properties. As acetaldehyde-induced DNA lesions produced under our experimental conditions decomposed at high temperature, primer/template complexes for DNA replication assay were assembled first and treated with acetaldehyde (Fig. 3a). When non-treated DNA templates were used, the polymerases synthesized DNA in a time-dependent manner (Fig. 3b, lanes 1–4 and Fig. 3c blue label). And since various random primers bound one pBSII template and form the complexes the start products by phi29 DNA polymerase were hardly detected (Fig. 3b, lane 1). In contrast, DNA polymerases did not produce new DNA from acetaldehyde-treated DNA templates (Fig. 3b, lanes 5–8 and Fig. 3c red label), suggesting that acetaldehyde-induced DNA lesions blocked replication reactions by phi29 DNA polymerase.

### RNA transcription reaction in acetaldehyde-treated plasmids

**Fig. 4:**
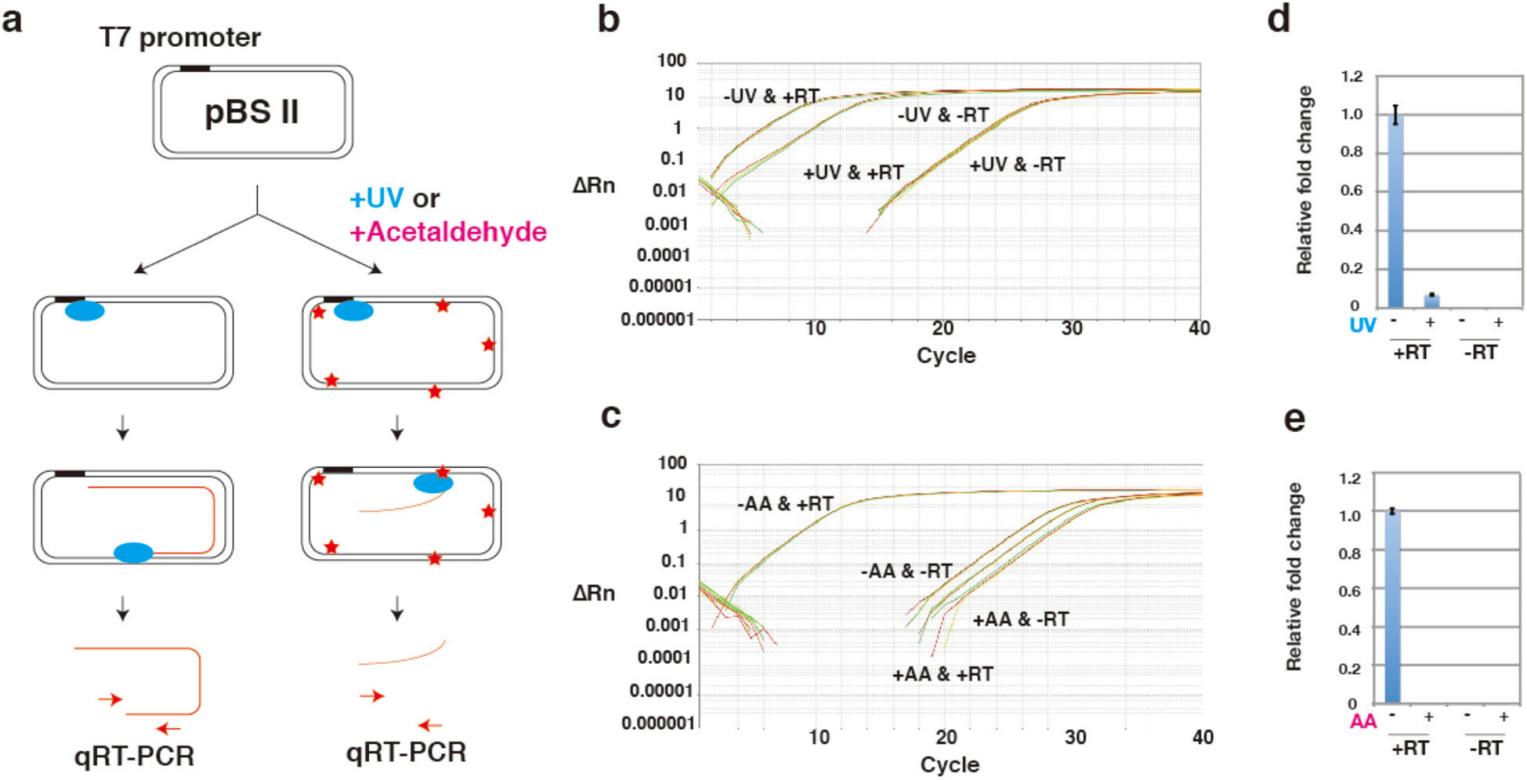
RNA transcription reaction of acetaldehyde-treated plasmids. **a** In the absence of DNA damage, the T7 RNA polymerase generates RNA transcripts from DNA templates. After purifying RNA, real-time reverse transcription-PCR (qRT-PCR) is performed, and the PCR products are analyzed. If acetaldehyde damages DNA, the resulting lesions inhibit RNA synthesis, as T7 RNA polymerase cannot synthesize transcripts from damaged templates, and qRT-PCR products will not be detected. Amplification plot of qRT-PCR analysis of RNA transcripts of UV-irradiated (**b**) or of acetaldehyde (AA)-treated (**c**) DNA templates. UV-irradiated (**d**) or acetaldehyde-treated (**e**) pBSII was incubated with T7 RNA polymerase, and transcription was quantified by qRT-PCR

Previously, we reported an in vitro method for detecting effects of chemically induced DNA lesions using in vitro transcription with T7 RNA polymerase and real-time reverse transcription polymerase chain reaction (PCR), based on inhibition of in vitro RNA synthesis (Fig. 4a) [15]. This assay was used for acetaldehyde. In this assay, we used UV-induced DNA lesions as a control condition. As expected, T7 transcription from UV-irradiated plasmids was inhibited, presumably by stalling of polymerase at DNA lesions (Fig. 4b and d). In contrast, transcription was not detected in acetaldehyde-treated plasmids (Fig. 4c and d), suggesting that transcription by T7 RNA polymerase was blocked in acetaldehyde-induced DNA lesions.

## Discussion

Previously, we reported that acetaldehyde forms reversible intrastrand GG crosslinks [14]. These crosslinks appear to be bulky DNA lesions, such as CPD and 6-4 pp. Plasmid DNA was treated with acetaldehyde under the aforementioned experimental conditions. Acetaldehyde-treated plasmid DNA remained incomplete digested by GG sequence-recognizing restriction enzymes, but was digested by non-GG sequence-recognizing restriction enzymes. Digestion of T4 DNA polymerase indicated that the acetaldehyde-induced DNA lesions were resistant to exonucleolytic activity. In treated plasmid DNA, neither DNA nor RNA synthesis reactions were observed. The effects of acetaldehyde on DNA metabolism may help explain the toxicity and mutagenicity of acetaldehyde.

As a highly reactive compound, acetaldehyde is believed to cause a variety of DNA lesions in living cells. Matsuda et al., reported that acetaldehyde induced GG to TT transversion mutations in the reporter *rpsL* gene in nucleotide excision repair (NER)-deficient XP-A cells, but not in normal cells [19]. This suggests that acetaldehyde forms NER-repairable GG lesions. As observed, acetaldehyde-induced GG dimers were resistant to digestion by restriction enzymes, suggesting that the GG dimers in DNA are bulky type lesions.

The direct products of the reaction between acetaldehyde and deoxyguanosine are considered to be Schiff base type adducts, such as *N*^2^-ethylidenedeoxyguanosine [20, 21]. This product can be stabilized by chemical reduction of the Schiff base to the stable product, *N*^2^-ethyldeoxyguanosine. Lesions interfere with RNA transcription reactions [22], but do not interfere with DNA replication reactions [23]. However, DNA replication was not observed in acetaldehyde-treated DNA templates. Therefore, it is possible that acetaldehyde-induced GG dimers produced under our experimental conditions may interfere with DNA replication. However, we cannot rule out the possibility that acetaldehyde-induced interstrand crosslinks also inhibited the replication reaction [13, 20]. Notably, guanine NH_2_ groups in crosslinked GG appear to be occupied by acetaldehyde. Thus, typical DNA polymerases may not incorporate cytosine opposite these lesions, resulting in single and/or double strand breaks. However, translesion DNA polymerases such as Pol eta may bypass these lesions and incorporate dATP opposite a guanine, as only two hydrogen bonding sites are available, ultimately resulting in GG-to-TT mutations [19].

Considering the effect of acetaldehyde-induced DNA lesions on DNA metabolism, these lesions should be repaired in living cells. However, studies indicating removal of such DNA lesions via DNA repair pathways are lacking. Most typical DNA lesions induced by acetaldehyde *N*^2^-ethyldeoxyguanosine appear to go unrepaired. Thus, we would like to propose one possibility that acetaldehyde-induced GG dimers produced under our experimental conditions are repaired by NER. Furthermore, since GG dimers interfere with RNA polymerase, transcription-coupled NER may be involved in removing GG dimers.

In the experimental conditions, we used a very high concentration of acetaldehyde (1 M) to analyze the effects of DNA lesions. Therefore, there are clear differences in between the experimental conditions and physiological conditions. As previously reported [14], however, acetaldehyde forms reversible intrastrand crosslinks in GG. In any case, we think that even low concentrations of acetaldehyde, such as those typically found in the human body, may induce genomic DNA lesions because the reaction is reversible.

Compared with our observation of acetaldehyde-induced GG dimers, other researcher reported that the sequence specificity of acetaldehyde was relatively low with respect to the damage induction [24]. There seem to be some discrepancies. We think, however, the difference from the acetaldehyde treatments in Methods. The researcher employed reduction agents that decompose GG dimers to detect more stable DNA lesions, *N*^2^-ethyldeoxyguanosine. Therefore, as far as they use this reduction agents, we think that they never observe the acetaldehyde-induced GG dimer.

## Conclusions

Acetaldehyde-treated DNA remained incomplete digested by GG sequence-recognizing restriction enzymes and were resistant to the exonucleolytic activity of T4 DNA polymerase. In addition, neither DNA nor RNA synthesis reactions were observed in acetaldehyde-treated DNA. The results suggested that acetaldehyde induces DNA lesions that interfere with DNA metabolism and may help explain the toxicity and mutagenicity of acetaldehyde.

## Data Availability

Not applicable

## Abbreviations

6-4 pp: pyrimidine (6–4) pyrimidone photoproduct
CPD: cis-syn cyclobutane pyrimidine dimer
GG: dGpdG
NER: nucleotide excision repair
pBSII: pBluescript II SK (−)
qRT-PCR: real-time reverse transcription-PCR
UV: Ultraviolet
